# Effective Adsorption of Patulin from Apple Juice by Using Non-Cytotoxic Heat-Inactivated Cells and Spores of *Alicyclobacillus* Strains

**DOI:** 10.3390/toxins10090344

**Published:** 2018-08-25

**Authors:** Marina Sajid, Sajid Mehmood, Chen Niu, Yahong Yuan, Tianli Yue

**Affiliations:** 1 College of Food Science and Engineering, Northwest A&F University, Yangling 712100, China; msajid1118@nwsuaf.edu.cn (M.S.); niuchen@nwsuaf.edu.cn (C.N.); yuan324@msn.com (Y.Y.); 2 State Key Laboratory of Crop Stress Biology for Arid Areas, College of Plant Protection, Northwest A&F University, Yangling 712100, China; sajid.mehmood@nwsuaf.edu.cn

**Keywords:** adsorption, apple juice, mycotoxin patulin, cytotoxicity, *Alicyclobacillus* cells and spores

## Abstract

Patulin (PAT) is a major threat to many food products, especially apple and apple products, causing human health risks and economic losses. The aim of this study was to remove PAT from apple juice by using the heat-inactivated (HI) cells and spores of seven *Alicyclobacillus* strains under controlled conditions. The HI cells and spores of seven strains adsorbed PAT effectively, and the HI cells and spores of *Alicyclobacillus acidocaldarius* DSM 451 (A51) showed maximum PAT adsorption capacity of up to 12.6 μg/g by HI cells and 11.8 μg/g by HI spores at 30 °C and pH 4.0 for 24 h. Moreover, the PAT adsorption process followed the pseudo-first order kinetic model and the Freundlich isotherm model; thermodynamic parameters revealed that PAT adsorption is a spontaneous exothermic physisorption process. The results also indicated that PAT adsorption is strain-specific. The HI cells and spores of *Alicyclobacillus* strains are non-cytotoxic, and the bioadsorption of PAT did not affect the quality of the juice. Furthermore, the cell wall surface plays an important role in the adsorption process.

## 1. Introduction

**Figure 1:**
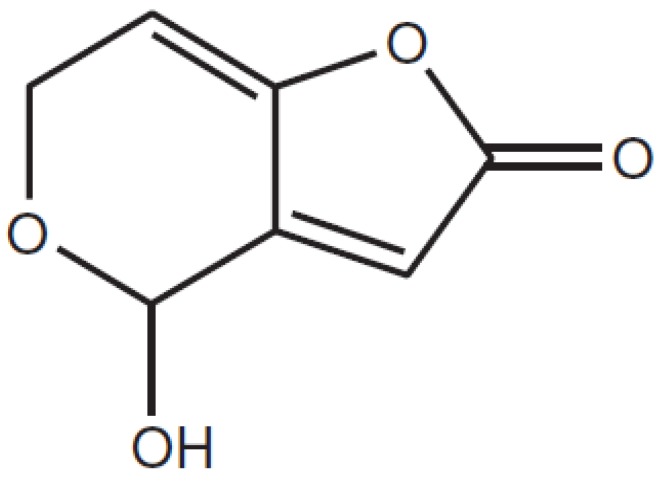
Chemical structure of patulin (PAT).

Patulin (PAT), 4-hydroxy-4*H*-furo[3,2-c]pyran-2(6*H*)-one is one of the most toxic secondary metabolites produced by the species of the genera *Aspergillus*, *Penicillium*, and *Byssochlamys* (Figure 1) [1]. *Penicillium expansum* is the main producer of PAT among these fungi, and it causes infection in a large number of fruits and fruit products [2]. The contamination of apple juice by PAT is one of the most important food safety issues worldwide [3]. The fungal species that are responsible for PAT production enter into the fruits through bruised and ruptured skin sites, and cause contamination [4]. PAT has caused several chronic health effects on genetics, immunity, and the central nervous system in animals, while its effects on humans are not clear yet [5]. The Joint FAO/WHO Expert Committee on Food Additives has set a maximum tolerable daily intake of 0.4 μg of PAT per kilogram of body weight [6], while the European Union has set a maximum of 50 μg of PAT for one liter of fruit juice [7].

Many physical and chemical approaches have been developed for the detoxification of PAT in apple juice and apple products. However, some disadvantages such as safety issues, possible losses in the nutritional quality, chemical hazards, limited efficacy, and high costs have been recorded for these approaches [8,9,10]. In addition to chemical and physical approaches, there has been a recent interest in the use of biological methods to remove PAT from fruit juices, particularly biosorption by using inactivated microbial cells [11,12].

Inactivated microorganisms with a large surface area, adsorptive selectivity, and abundant functional groups are in demand as adsorbents. Due to the advantages of biosorption, numerous microorganisms such as lactic acid bacteria (LAB) [12,13,14,15,16,17], *Gluconobacter oxydans* [18], *Saccharomyces cerevisiae* [19,20], and *Alicyclobacillus* spp. [21], have been reported for the adsorption and removal of PAT. The adsorption of PAT in apple juice by using 10 LAB strains was influenced by the initial concentration of toxin and temperature. Meanwhile, Fourier-transform infrared spectroscopy (FTIR) results suggested that the cell wall plays an important role in PAT adsorption [14]. Wang et al. [17] suggested that the heat-inactivated (HI) LAB cells were more likely to have a higher capacity to adsorb PAT from the aqueous solution. Guo et al. [20] found similar efficiencies of two types of inactivated yeast powder: laboratory-prepared yeast powder (LYP) and commercial yeast powder (CYP) to adsorb PAT from apple juice, and suggested that inactivated yeast powder had potential as a novel and promising adsorbent to bind PAT effectively. Yuan et al. [21] for the first time used the inactivated cells of 12 *Alicyclobacillus* spp. to reduce PAT from apple juice. Their results provided a theoretical foundation for the recycling of *Alicyclobacillus* cells from spoiled apple juice.

So far, no studies concerning the PAT adsorption mechanism by HI cells and spores of *Alicyclobacillus* strains have been reported. Moreover, the potential influence of HI cells and spores of *Alicyclobacillus* strains used for PAT adsorption on the environment and human health need to be considered. The objectives of this study were to investigate the potential abilities of HI cells and the spores of seven *Alicyclobacillus* strains to reduce the level of PAT in apple juice. The cytotoxicity test was conducted to evaluate the possible toxicity of HI cells and spores of *Alicyclobacillus* strains using FTIR. The factors affecting PAT adsorption rate, including pH, temperature, contact time, and initial PAT concentration, were also studied. Two adsorption models (kinetics and equilibrium isotherms) with thermodynamic parameters were applied to fit the experimental data.

## 2. Results

### 2.1. Adsorption of PAT by HI Cells and Spores of Seven Alicyclobacillus Strains

**Figure 2:**
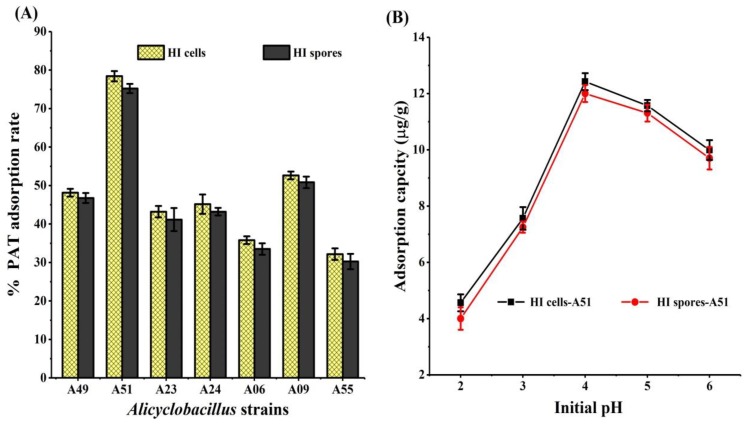
(**A**) Percentage adsorption rate of PAT (200 μg/L) by the heat-inactivated (HI) cells and spores of seven *Alicyclobacillus* strains (1.0 g) from apple juice at 30 °C, pH 4.0, and 24 h of incubation time. The bars show the mean value of three replicates; error bars indicate the standard deviation, and are significantly different based on one-way analysis of variance (ANOVA) (*P* < 0.05). (**B**) Effect of initial pH on the adsorption of PAT by the HI cells and spores of *Alicyclobacillus acidocaldarius* DSM 451 (A51).

HI cells and spores of seven *Alicyclobacillus* strains had shown different PAT adsorption abilities (Figure 2A). However, the differences among the spores and cells of different strains were highly significant; only the differences between spores and cells of the same strain were not significant (*P* > 0.05). The highest adsorption rates—78.41% and 75.20%—were observed by the HI cells and spores of A51, which were two times greater than those of the least-efficient HI cells and spores of A55, respectively, as shown in Figure 1A. After treatment with the HI cells and spores of *Alicyclobacillus* strains, different quality parameters of apple juice were assessed (Table 1). No significant differences (*P* > 0.05) were found in the values for degrees Brix, total sugar, titratable acidity, color value, and clarity between the treated juice and control samples, except for the HI cells and spores of A06. On the basis of the remarkable superiority of biomass (Figure S1) and PAT adsorption rate, the HI cells and spores of A51 were regarded as the most efficient PAT binders and subjected to study further.

### 2.2. Effect of Incubation Conditions on PAT Adsorption

**Figure 3:**
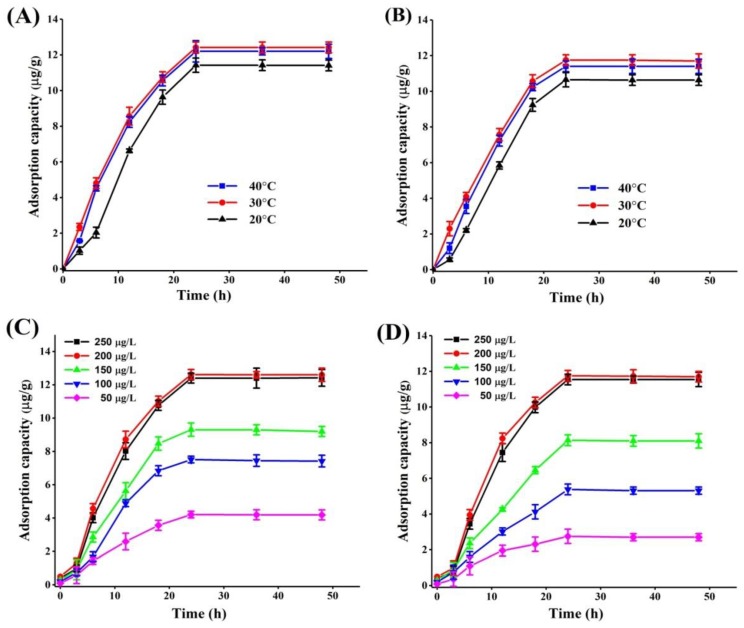
Effect of different initial temperatures on the adsorption kinetics of patulin (PAT) by the (**A**) heat-inactivated (HI) cells and (**B**) HI spores of A51. Effect of different initial PAT concentrations on the adsorption kinetics of PAT by the (**C**) HI cells and (**D**) HI spores of *Alicyclobacillus acidocaldarius* DSM 451 (A51).

The amount of PAT adsorption was the highest at pH 4.0 (Figure 2B). The data demonstrated that the adsorption capacity increased significantly between pH 2.0–4.0; later, it decreased with an increment of pH. With an increased temperature from 20 °C to 30 °C, PAT adsorption increased from 11.4 μg/g to 12.6 μg/g by the HI cells of A51, and from 10.7 μg/g to 11.8 μg/g by HI spores of A51. With a further increase in temperature from 30 °C to 40 °C, there was no significant increase in PAT adsorption capacity by both HI cells and spores (Figure 3A, B). PAT adsorption increased from 4.2 μg/g to 12.6 μg/g by HI cells (Figure 3C) and from 2.8 μg/g to 11.8 μg/g by HI spores (Figure 3D) with the increase of initial PAT concentration of 50 μg/g to 200 μg/g, which then slowed as it approached equilibrium.

### 2.3. Cytotoxicity Test

**Figure 4:**
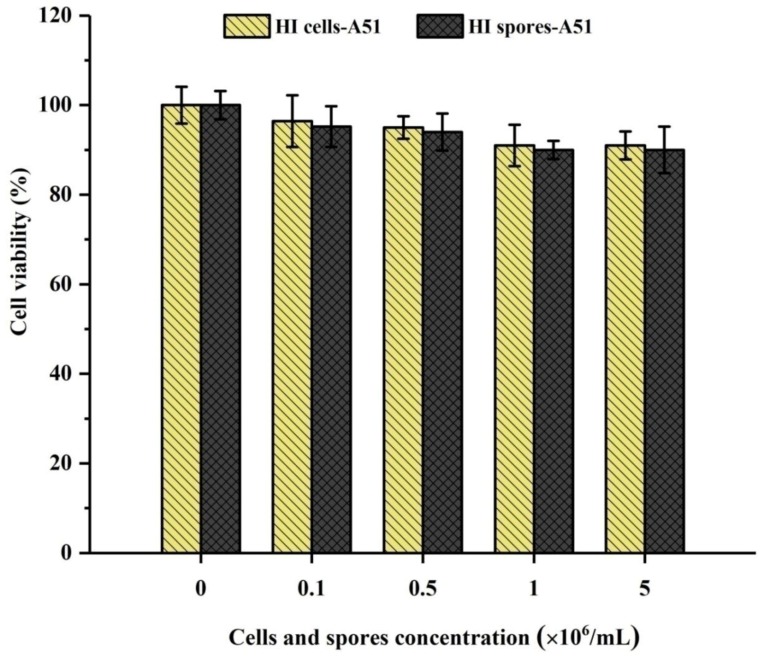
The cytotoxicity of the Heat-inactivated (HI) cells and spores of *Alicyclobacillus acidocaldarius* DSM 451 (A51) prepared with different concentrations of active cells and spores of strain A51 by using HepG2 cells.

The possible in vitro cytotoxicity evaluation by methyl thiazol tetrazolium bromide (MTT) assay was assessed by HI cells and spores of A51 with different concentrations of active cells and spores. The results showed a gradual decrement trend of cells viability with 0.1 × 10^6^/mL to 5 × 10^6^/mL concentration of active cells and spores used for the preparation of HI cells and spores. HepG2 cells after 24 h of exposure to HI cells and spores showed no significant cytotoxicity, and the cell viability was still higher than 80%, as shown in Figure 4. In addition, the cytotoxicity grading was qualified, as described in Table S1.

### 2.4. FTIR Analysis

**Figure 5:**
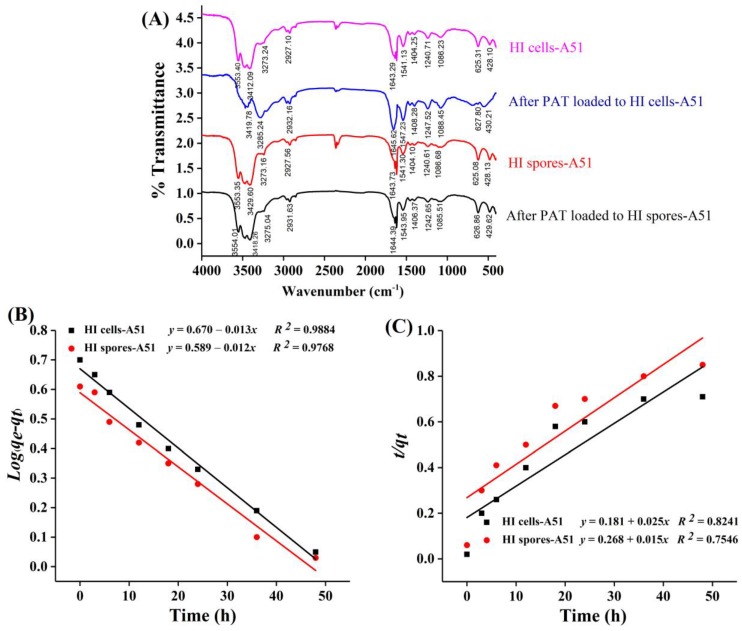
(**A**) Fourier-transform infrared spectroscopy (FTIR) spectra for the heat-inactivated (HI) cells and spores of *Alicyclobacillus acidocaldarius* DSM 451 (A51) before and after PAT absorption. The pseudo-first order (**B**) and the pseudo-second order (**C**) kinetics model of PAT adsorption by the HI cells and spores of A51, respectively.

The FTIR spectra of HI cells and spores of A51 before and after PAT uptake are shown in Figure 5A. The adsorption peaks at 3553 cm^−1^, 3412 cm^−1^, 3273 cm^−1^ (O–H/N–H symmetrical and asymmetrical stretching vibrations), 2927 cm^−1^ (C–H/–CH_2_ symmetrical stretching vibration), 1643 cm^−1^ (stretching band of C=O from the amide I), 1541 cm^−1^ (stretching band of COO–), 1404 cm^−1^(–SH/C–N from amide II bending vibration), 1240 cm^−1^ (the C-N from amide III), 1086 cm^−1^ (the asymmetrical bending vibration of polysaccharides O–C bonds), 625 cm^−1^ (alkyl halide C–Br stretching vibrations) and 428 cm^−1^ (alkyl halide C–I bending vibration) were observed in the biomass spectra of HI cells and spores of A51 before PAT uptake.

After PAT was adsorbed onto HI cells and spores, the wavenumbers and intensities of some peaks were shifted with some changes compared with those before absorption (Figure 5A). The peak at 3553 cm^−1^ disappeared for the HI cells of A51. Meanwhile, the –OH group of the HI cells and spores of A51 revealed a clear shift to a higher frequency at 3419 cm^−1^ and 3429 cm^−1^, respectively. The evident changes of adsorption peaks at 3285 cm^−1^ by HI cells and 327 cm^−1^ by HI spores inferred the involvement of the N–H group. Increasing peak intensity at 1645 cm^−1^ and 1644 cm^−1^ for HI cells and spores, respectively, showed that the C=O amide groups were involved in the PAT adsorption. The peak at 1404 cm^−1^ that corresponds to –SH vibrations shifted to higher wave numbers. It is observed that the FTIR spectra of HI cells and spores loaded with PAT had some changes compared with those of the natural one. The results of FTIR analysis showed that many of the functional groups, such as C=O, COO–, P–O–C, N–H, and –SH groups, were involved onto the cell wall surfaces of HI cells and spores of A51 for the PAT adsorption process.

### 2.5. Kinetic and Equilibrium Isotherm Modeling

#### 2.5.1. PAT Adsorption Kinetics

To understand the kinetics mechanism that controls the adsorption processes such as mass transfer and chemical reactions, the pseudo-first order kinetic and pseudo-second order kinetic equations were used for PAT adsorption onto the HI cells and spores of *Alicyclobacillus* strains.

Pseudo-first order kinetic rate equation:

(1) $$\mathrm{Log}\left(q_{e}-q_{t}\right)=\log q_{e}-\frac{k_{f}}{2.303}$$

Pseudo-second order kinetic rate equation:

(2) $$\frac{t}{q_{t}}=\frac{1}{k_{s}q_{e}^{2}}+\frac{1}{q_{e}}t$$

where *k_f_* is the first-order kinetic rate constant; *q_e_* (μg/g) and *q_t_* (μg/g) are the amounts of adsorbed PAT at equilibrium time (h) and at a given time (*t*), respectively; and *k_s_* is the second-order kinetic rate constant. 

The fitted trends of the pseudo-first order kinetic and the pseudo-second order kinetic are shown in Figure 5B, C. The fitting results of the kinetic parameters for HI cells and spores of A51 are shown in Table 2. The *R*^2^ values of the pseudo-first order adsorption kinetics rate were higher than those of the pseudo-second order kinetic rate for PAT adsorption. The calculated equilibrium adsorption capacity (*q_e,cal_*) in the pseudo-first order kinetic rate fitted the experimental values (*q_e_*) well.

#### 2.5.2. PAT Adsorption Isotherms

The Langmuir and Freundlich isotherm models were used to analyze the surface properties of the adsorbent. 

Langmuir isotherm model:

(3) $$\frac{C_{e}}{q_{e}}=\frac{1}{q_{m}K_{L}}+\frac{C_{e}}{q_{m}}$$

Freundlich isotherm model:

(4) $$\mathrm{Ln}\;q_{e}=\ln K_{F}+\frac{1}{n}\ln C_{e}$$

where $q_{m}$ and $q_{e}$ are the monolayer highest adsorption capacity and adsorption capacity at equilibrium (μg/g), respectively; $K_{L}$ is the Langmuir constant related to the energy of adsorption and affinity of the binding sites; *c_e_* (μg/L) is the equilibrium concentration of PAT in apple juice; $K_{F}$ and 1/n are the Freundlich constants related to the adsorption capacity and adsorption intensity (dimensionless), respectively; and 1/n is an indication of the favorability of adsorption (1/n > 1: unfavorable; 0 < $R_{L}$ < 1: favorable; 1/n= 1:irreversible) [22,23].

**Figure 6:**
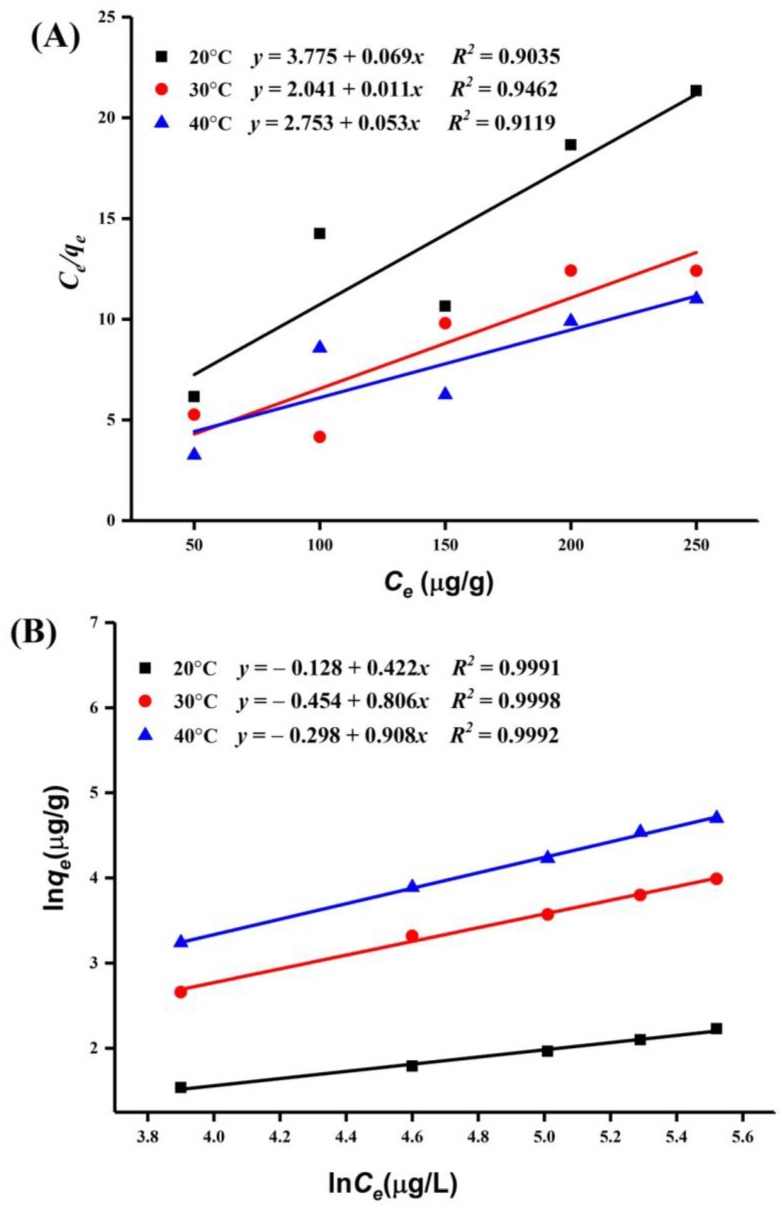
Langmuir (**A**) and Freundlich(**B**) isotherm models of patulin (PAT) adsorption by the heat-inactivated (HI) cells of *Alicyclobacillus acidocaldarius* DSM 451 (A51).

**Figure 7:**
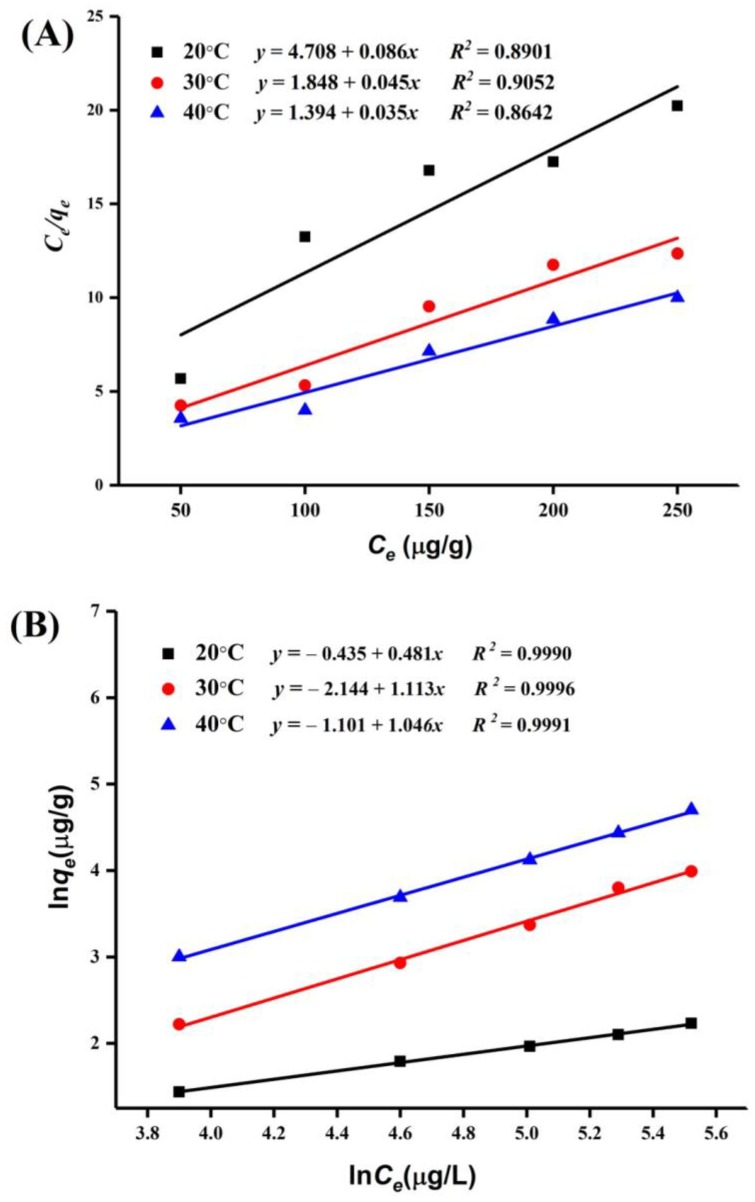
Langmuir (**A**) and Freundlich (**B**) isotherm models of PAT adsorption by the heat-inactivated (HI) spores of *Alicyclobacillus acidocaldarius* DSM 451 (A51).

The PAT adsorption equilibrium data on the HI cells and spores of A51 are shown in Table 3. We found that the data fit better with the Freundlich isotherm model than the Langmuir isotherm model (Figure 6 and Figure 7). It suggests that the PAT adsorption process on the HI cells and spores of A51 was based on multilayer adsorption. The adsorption intensity (1/n) values demonstrated that PAT adsorption was more favorable at 30 °C.

### 2.6. Thermodynamics Parameters

The adsorption thermodynamic parameters such as the Gibbs free energy change [($\Delta G^{{}^{\circ}}$, KJ/mol], enthalpy change [($\Delta H^{{}^{\circ}}$, KJ/mol], and entropy change [$\Delta S^{{}^{\circ}}$, J/mol·K], were calculated by using the following van’t Hoff equations, in order to describe what processes occurred spontaneously. The experiments were conducted at three different temperatures (293 K, 303 K, and 313K).

(5) $$\ln K_{F}=\frac{-\Delta H^{{}^{\circ}}}{RT}+\frac{\Delta S^{{}^{\circ}}}{R}$$

(6) $$\left(\Delta G^{{}^{\circ}}\right)=\Delta H^{{}^{\circ}}-T\Delta S^{{}^{\circ}}$$

where R [8.314 (J/mol·K)] is the gas constant; and T is the absolute temperature (K). The values of $\Delta H^{{}^{\circ}}$ and $\Delta S^{{}^{\circ}}$ can be calculated by the slope and intercept obtained by $\ln K_{F}-1/T$ fitting.

The results obtained for $\Delta G^{{}^{\circ}}$ were −27.10 KJ/mol, −27.12 KJ/mol, and −27.97 KJ/mol by the HI cells of A51, and −22.25 KJ/mol, −22.27 KJ/mol, −22.89 KJ/mol by the HI spores of A51. The decrease of ΔG° values with the increase of temperature indicated that the adsorption became less at a higher temperature, and it was a spontaneous process. The increased temperature promoted the mobility of PAT molecules from the solid phase to the liquid phase. The calculated $\Delta H^{{}^{\circ}}$ for the HI cells and spores were found to be −7.42 KJ/mol and −5.86 KJ/mol, respectively. Meanwhile, the results of $\Delta S^{{}^{\circ}}$ for the HI cells and spores of A51 were 20.56 J/(mol·K) and 19.95 J/(mol·K), respectively. The negative value of $\Delta H^{{}^{\circ}}$ indicated that the adsorption process was exothermic. In addition, the positive value of $\Delta S^{{}^{\circ}}$ corresponds to an increased randomness at the solid–liquid interfaces during the adsorption of PAT [24] onto the HI cells and spores of A51 with good affinity.

## 3. Discussion

This study demonstrates that the HI cells and spores of seven *Alicyclobacillus* strains were able to remove PAT from apple juice at significantly different adsorption rates (Figure 2A). The highest PAT adsorption (12.6 μg/g and 11.8 μg/g) was observed by the HI cells and spores of A51, (Figure 3C,D). These results are in good agreement with previous reports that the PAT adsorption capability of the HI microorganism was strain-dependent. The differences in the PAT adsorption abilities of the HI cells and spores of *Alicyclobacillus* strains are due to their distinct cell wall compositions and different organizations within the cell structures [12,21].

We identified that the A51 strain has the highest PAT adsorption capacity among seven tested strains. Hatab et al. [14] identified two strains with high detoxifying capacities, namely the LR5 and EF10, which removed 80.4% and 64.5% of PAT from apple juice, respectively. The inactivated microbial cells of LAB and yeasts have previously been reported to reduce PAT from apple juice and aqueous solutions [12,13,17,21]. The adsorption of PAT by the HI cells and spores of all of the tested strains have not shown any negative impact on the quality of the apple juice based on the measurements of various quality parameters except for the HI cells and spores of *Alicyclobacillus* *cycloheptanicus* DSM 4006 (A06) (Table 1). Similarly, it has been reported earlier that juice samples that had been treated with microorganisms had not significantly affected the quality of the treated apple juice [14,21,25]. Yuan et al. [21] used 12 different inactivated yeast strains to remove PAT from juice, and they found that the quality of the treated and untreated apple juice was very similar. Similarly, Hatab et al. [14] also used 10 different strains of the inactivated LAB to remove PAT from apple juice, and no significant differences were found in the quality parameters between treated and non-treated apple juices. However, the different results obtained by Fathi-Achachlouei et al. [26], who reported that activated carbon (AC) removed PAT considerably from apple juice. Although it is a very efficient and time-saving approach, it has negative impacts on the quality of the apple juice, affecting its color, clarity, acidity, total sugar, pH, and degrees Brix. Moreover, the results in this study indicated that the HI cells and spores of A51 were biocompatible with negligible cytotoxicity (Figure 4). The PAT adsorption capacities of different adsorbents are listed in Table S2.

The incubation time, pH value, initial toxin concentration, and temperature of the reaction solution play an essential role during the PAT-binding process, which significantly influences both the surface characteristics of the adsorbent and chemical properties of adsorbate. We found that the PAT adsorption rate by the HI cells and spores of A51 was highest at pH 4.0 (Figure 2B) and 30 °C (Figure 3A,B) for 24 h (Figure 3C,D). These results are in agreement with Topcu et al. [12], who used two *Enterococcus faecium* strains to remove PAT from aqueous solution, and found that the removal of PAT was highest at pH 4.0. Similarly, Peng et al. [27] used cross-linked xanthated chitosan resin (CXCR) for the effective biosorption of PAT from apple juice, and showed that the optimum adsorption conditions of CXCR for PAT was achieved at pH 4.0 and 30 °C for 18 h. Yuan et al. [21] found that the PAT removal percentages for *AAT92* and *AAT96* significantly (*P* < 0.05) increased with extended incubation time from 16.6% (1 h) to 55.0% (24 h), and from 11.4% (1 h) to 60.2% (24 h), respectively.

A further increase of temperature from 30 °C to 40 °C reduced the adsorption of PAT. Topcu et al. [12] found a reduction of PAT adsorption by the nonviable cells of two strains of *Enterococcus faecium* with a further increase in incubation time from 16.1% (1 h) to 38.6% (48 h) and from 19.7% (1 h) to 36.4% (48 h), respectively. We also observed that the adsorption capacity increased rapidly with the increase of initial PAT concentration, and then slowed as it reached the maximum value of apple juice. It revealed that the rapid initial adsorption rate decreased at an initial PAT concentration of 250 μg/g; however, no significant differences appeared between the HI cells and spores of A51 due to the rapid attachment of molecules to the surface. Hatab et al. [14] found that the biosorption capacity of PAT was influenced by incubation temperature and the initial concentration of toxin. Yuan et al. [21] found the maximum removal rate of PAT with an initial toxin concentration of 100 μg/L, showing a significant difference (*P* < 0.05) from that of 200 μg/L and 300 μg/L for two selected *Alicyclobacillus* spp. Similarly, Fuchs et al. [16] found a significantly decreased PAT reduction rate by the LAB when PAT concentration was raised from 1 μg/L to 100 μg/L. The decreasing trend of PAT adsorption is possibly due to the initial PAT concentration, as suggested by Hatab et al. [13], Guo et al. [20], and Yuan et al. [21], who reported an increase in adsorption ability with an increase of time to remove PAT. Similarly, Peng et al. [27] showed that the adsorption capacity increased rapidly with the increase of initial PAT concentration, and then slowed as it reached the maximum value.

FTIR results confirmed that the compounds responsible for PAT adsorption onto the HI cells and spores of A51 were the cell wall components (carboxyl, polysaccharides, hydroxyl, lipids, and amino). Similarly, it has been previously reported that polysaccharides and peptidoglycans on the cell wall surface were the main components for the binding of mycotoxins [13,14,17,25]. The results of kinetic studies showed that the pseudo-first order adsorption kinetic model was a more suitable model for predicting the PAT adsorption reaction onto the HI cells and spores of A51, which suggests that the biosorption process is controlled by the diffusion process [28,29]. Moreover, Qiu et al. [29] reported similar results: that the kinetics of PAT adsorption by nano-Fe_3_O_4_ modified inactivated yeast followed a pseudo-first order adsorption kinetics model. Meanwhile, the thermodynamic parameters showed that the adsorption of PAT onto the HI cells and spores of A51 was a spontaneous and exothermic process. These results are in good agreement with Yue et al. [25], Qiu et al. [29], Schiewer et al. [30], and Luo et al. [31].

Well-fitted equilibrium data that were explained by the Freundlich isotherm model represent the non-ideal adsorption on heterogeneous surface energy systems, which corresponds with the rough and heterogeneous surface structure of the HI cells and spores of A51. Our findings are in agreement with the results of Yue et al. [25], who reported that the Freundlich model is more suitable for the adsorption of PAT onto Ca-alginate-AC beads. However, different results were observed by Peng et al. [27], who reported that the Langmuir model is more suitable for the adsorption of PAT.

## 4. Conclusions

*Alicyclobacillus* specific strains have the ability to remove PAT from apple juice very effectively without any negative effects on juice quality. The adsorption rate is affected by the incubation conditions and specific properties of the cell wall surface. This study provides a first experimental insight into the kinetic mechanism and isotherm behavior of the HI cells and spores of *Alicyclobacillus* strains to remove PAT from apple juice. Further studies are needed to make the availability of potential biosorbent *Alicyclobacillus* strains at a commercial level to remove PAT from apple juice.

## 5. Materials and Methods

### 5.1. Materials

#### 5.1.1. Chemicals and Solvents

Standard PAT in crystalline form, methanol, and acetonitrile, were obtained from Sigma-Aldrich (St. Louis, MO, USA). Ultrapure water was prepared by using a Milli-Q water purification system (Milli-pore, Bedford, MA, USA). *Alicyclobacillus acidocaldarius* medium (AAM) broth, AAM agar, phosphate-buffered saline (PBS), methyl thiazol tetrazolium bromide (MTT), RPMI-1640 media, and dimethylsulfoxide (DMSO) were obtained from Solarbio Science & Technology Co. Ltd. Beijing, China. Human hepatocellular carcinoma HepG2 cell lines were obtained from the Department of Preventive Veterinary, College of Veterinary Medicine, Northwest A&F University, Yangling, Shaanxi, China. All of the other chemicals and solvents were purchased from a local chemical reagents company in Yangling, Shaanxi, China.

#### 5.1.2. Bacterial Strains

Seven standard strains of Alicyclobacillus species viz: Alicyclobacillus acidocaldarius (DSM 449, DSM 451), Alicyclobacillus acidoterrestris (DSM 3923, DSM 3924), Alicyclobacillus cycloheptanicus (DSM 4006), Alicyclobacillus herbarius (DSM 13609), and Alicyclobacillus pomorum (DSM 14955), were obtained from German Resource Centre for Biological Material (DSMZ). All of the strains were coded separately (Table S3). To prevent any change in the cell membranes, the strains were preserved in 30% (*w*/*v*) glycerin at −40 °C.

#### 5.1.3. Apple Juice

The PAT-free concentrated apple juice was purchased from Hengxing Juice Company (Baoji, Shaanxi, China) with a soluble content of 70 Brix. The diluted apple juice (12 °Brix, pH 4.0) was filtered and sterilized at 100 °C for 10 min in caped flasks. PAT standard stock solution at a concentration of 100 mg/L was prepared in an organic solvent and stored at −40 °C. To obtain PAT-contaminated apple juice (200 μg/L), the organic solvent ethyl acetate was evaporated from PAT standard stock solution using a rotary evaporator at 45 °C to dryness, and the residue was immediately suspended in sterile diluted apple juice.

### 5.2. Preparation of HI Alicyclobacillus Cells and Spores

*Alicyclobacillus* strains were cultured separately in sterile AAM broth and incubated on a shaker (Fine mixer SH2000 orbital shaker, Gunpo-si, Korea) at 120 rpm for 24–48 h with their specific growing conditions and enriched media (Table S3). The cultivated cells were harvested by centrifugation (3600× *g*, 20 min at 4 °C) and washed three times with distilled water. Active bacterial cell count was done using the plate counting method [32].

Sporulation was done as described by Torlak [33], with slight modifications. After incubation, sporulation was confirmed by microscopy following staining with 5% malachite green and 0.5% counter-stain (safranin). The spore suspensions were centrifuged (3600× *g*, 10 min) and washed three times with distilled water. The concentration of active spores of each strain, determined by plating 100 μL of appropriate dilutions onto AAM agar, was adjusted to 10^6^ spores/mL with distilled water. To obtain HI cells and spores, autoclaving was done at 121 °C for 20 min. The HI cells and spores of *Alicyclobacillus* strains were lyophilized and powdered using a mortar and pestle for adsorption experiments.

### 5.3. Batch Adsorption Experiments

The adsorption assays were carried out by adding 1.0 g of the weight of HI cells and spores of seven *Alicyclobacillus* strains into 60 mL of PAT-contaminated apple juice (200 μg/L) in 150-mL sterile conical flasks. The test solutions were thoroughly mixed and incubated on a shaker at 120 rpm for 24 h to allow PAT binding assay. After incubation, the supernatants of each treated juice were collected for the measurement of PAT levels (10 mL) and juice quality parameters (50 mL). A Millex syringe driven PTEF filter (0.2 μm) was used for purification before HPLC detection. Next, the samples were detected by HPLC with UV detection. PAT-contaminated apple juice without *Alicyclobacillus* strains was prepared and used as a control sample. All of the assays were performed in triplicate.

The effect of pH on the adsorption capacity of PAT was investigated within the pH range from 2.0 to 6.0. The effect of different initial PAT concentrations was investigated in the range of 50 μg/L to 250 μg/L. The effect of temperature was conducted at 20 °C, 30 °C, and 40°C. The effect of contact time was investigated at eight different levels for a total of 48 h (Table S4). The percentage of PAT adsorption rate and adsorption capacity ($q_{e})$ were calculated according to Equations (7) and (8):

(7) $$\%\;\mathrm{PAT}\;\mathrm{adsorption}\;\mathrm{rate}=\left(1-\left[\frac{\mathrm{PAT}\;\mathrm{peak}\;\mathrm{area}\;\mathrm{of}\;\mathrm{the}\;\mathrm{sample}}{\mathrm{PAT}\;\mathrm{peak}\;\mathrm{area}\;\mathrm{of}\;\mathrm{control}}\right]\right)\times 100$$

(8) $$q_{e}=\frac{\left(C_{i}-C_{f}\right)\times V}{M}$$

where $q_{e}$ is the adsorption capacity (μg/g) at time t; $C_{i}$ and $C_{f}$ are the initial and final concentrations of PAT (μg/L), respectively; V is the volume of PAT solution (mL); and M isthe weight (g) of the added HI cells and spores of *Alicyclobacillus* strains.

### 5.4. Quality Parameters of Apple Juice

The effect of the HI cells and spores of *Alicyclobacillus* strains on the juice quality parameters were assessed. °Brix of apple juice before and after treatment was determined by a hand-held refractometer (model 10430; Reichert, Depew, NY, USA). The total sugar of apple juice was calculated by a direct titrimetric process with Fehling’s reagent (SB/T 10203-1994). Titration determined titratable acidity (TA) by the titrimetric method in final apple juice with 0.1 N of NaOH and a phenolphthalein indicator (SB/T 10203-1994). To determine the juice clarity, the samples’ transmittance at 625 nm was measured by a spectrophotometer (UV2550, Shimadzu Scientific Instruments, Columbia, MD, USA). Juice color was measured using spectrophotometer at 440 nm (GB/T18963) [21].

### 5.5. In Vitro Cytotoxicity Evaluation

HepG2 cells were maintained and cultured according to the method described by Yu and Huang [34]. The cytotoxicity of the HI cells and spores of *Alicyclobacillus* strains was determined by MTT assay [35]. Initially, 100 μL of HepG2 cells (1 × 10^4^) were seeded in each well of a 96-well plate and maintained at 37 °C in an incubator (5% CO_2_ and 95% humidity) for 24 h. Next, the cells in each well were treated with 100 μL of fresh medium containing 200 μg/mL of HI cells and spores obtained from different concentrations of activated cells and spores of *Alicyclobacillus* strains. The supernatant was removed, and the cells were washed three times with PBS after 24 h. Then, the cells were cultured for 2 h at 37 °C with 100 μL of MTT solution (10% 5 mg/mL of MTT agent with 90% RPMI-1640 medium). After careful aspiration of MTT containing a medium, cells were dissolved in 100 μL of DMSO per well for 10 min. All of the samples were tested in triplicate. An iMark Microplate Reader was used to express the relative cell viability at a wavelength of 570 nm. HepG2 cells grown in medium without any treatment were taken as control.

### 5.6. Characterization

To assess the potential functional groups on the surface of the HI cells and spores of *Alicyclobacillus* strains, FTIR analysis was performed by using a Nicolet-740SX FTIR spectrophotometer (USA). The dry weight (1 mg) of the HI cells and spores of *Alicyclobacillus* strains were separately mixed and grounded with 100 mg of KBr (Spectral) in an agate mortar. After obtaining the background, 25–30 mg of each dry KBr sample mixture was pressed into a transparent disc. All of the infrared (IR) spectral range 4000–400 cm^−1^ was recorded at room temperature to observe the biomass before and after PAT loaded. All of the experiments were performed in triplicate.

### 5.7. Sample Extraction and Quantification of PAT

The AOAC international official method (2000.02) for the detection and quantification of PAT in clear and cloudy apple juices and apple puree was used for the extraction of PAT with slight modifications [27]. Test apple juice (10 mL) was extracted three times with 20 mL of ethyl acetate. The organic phase was collected and purified by 4 mL 1.5% (*w*/*v*) of sodium bicarbonate (NaHCO_3_) aqueous solution. After purification, the organic phase was dried with 15 g of anhydrous sodium sulfate and then distilled by a rotary evaporator at 45 °C. PAT residue in the distillation flask was resuspended in 1 mL of deionized water adjusted to pH 4.0 with acetic acid. HPLC analysis was conducted immediately after filtering through a 0.22-μm pore size membrane.

The LC-2010AHT HPLC system (Shimadzu Scientific Instruments, Columbia, MD, USA) with a UV absorbance detector and an Alltima reversed-phase column C18 (250 × 4.6 mm internal diameter, 5-mm particle size) was used to determine the residual PAT. The detection wavelength was set at 276 nm. A 20-μL sample or standard solution of PAT was in-poured, and HPLC grade acetonitrile/water (10:90, *v*/*v*) was used as the isocratic mobile phase with a flow rate of 1 mL/min at 40 °C. All of the calibration standards and samples with control were run in triplicate for each matrix. An HPLC method was validated by the adaptation of the method given by Yuan et al. [36]. The validation of this method was based on the criteria of linearity, reproducibility, limits of detection (LODs), limits of quantification (LOQs), recovery, and matrix effects. Under the HPLC conditions, the retention time of PAT was found to be about 7.00 ± 0.5 min. Linearity was confirmed by using the calibration curve for each PAT concentration given in Figure S2. It was linear in the range of 50–250 μg/L (50 μg/L, 100 μg/L, 150 μg/L, 200 μg/L, and 250 μg/L) with a correlation coefficient *R*^2^ = 0.999, indicating a good calibration curve. Reproducible standard curves were generated weekly, and detection levels were measured against prepared calibration standards. The LODs and LOQs were 5 μg/L and 15 μg/L, respectively. The average recovery value of the validation method was 96 ± 0.5%. Figure S3 shows a typical chromatogram 200 μg/L of PAT. As shown, there was a minimal matrix effect in the area of interest, and positive quantifications were highly reproducible and effective.

### 5.8. Statistical Analysis

All of the adsorption experiments were performed in triplicate; the results were presented as means ± standard deviation. Data were subjected to one-way ANOVA followed by Duncan’s multiple comparisons with IBM SPSS Statistics software 23.0 (IBM Corp., Armonk, NY, USA). Statistical significance was considered to exist at *P* < 0.05.

## Figures and Tables

**Table 1:** Quality parameters of apple juice treated with heat-inactivated (HI) cells and spores of seven *Alicyclobacillus* strains.

Strains or Matrix	Quality Parameters				
	°Brix	Total Sugar (g/100 mL)	Titratable Acidity (g/100 mL)	Color Value (%)	Clarity (%)
CK_1_*	12.58 ± 0.37	11.43 ± 0.04	0.34 ± 0.03	86.43 ± 1.67	95.43 ± 0.37
CK_2_* (Control)	12.75 ± 0.52	10.61 ± 0.06	0.33 ± 0.01	80.51 ± 2.74	93.17 ± 0.46
HI cells-A49	12.43 ± 0.56	10.79 ± 0.15	0.33 ± 0.03	81.57 ± 1.53	93.73 ± 1.73
HI spores-49	12.96 ± 0.26	11.27 ± 0.18	0.35 ± 0.05	80.32 ± 1.24	92.03 ± 2.34
HI cells-A51	12.60 ± 0.38	11.00 ± 0.03	0.33 ± 0.02	85.10 ± 3.48	95.73 ± 167
HI spores-A51	12.64 ± 0.46	10.86 ± 0.06	0.34 ± 0.08	85.90 ± 3.17	94.73 ± 0.62
HI cells-A23	12.85 ± 0.50	11.59 ± 0.09	0.34 ± 0.06	81.62 ± 2.21	96.07 ± 0.49
HI spores-A23	12.78 ± 0.50	10.64 ± 0.19	0.36 ± 0.02	81.34 ± 2.63	94.93 ± 0.16
HI cells-A24	12.72 ± 0.25	11.62 ± 0.08	0.35 ± 0.06	82.38 ± 1.81	95.79 ± 1.90
HI spores-A24	13.00 ± 0.12	11.75 ± 0.36	0.34 ± 0.02	88.42 ± 0.49	96.36 ± 0.86
HI cells-A06	13.00 ± 0.16	10.84 ± 0.38	0.33 ± 0.03	30.29 ± 1.59	53.97 ± 0.48
HI spores-A06	12.92 ± 0.58	11.72 ± 0.26	0.35 ± 0.01	36.44 ± 1.39	50.84 ± 1.09
HI cells-A09	12.86 ± 0.43	10.85 ± 0.06	0.36 ± 0.03	81.38 ± 2.47	93.16 ± 0.26
HI spores-A09	13.00 ± 0.53	10.79 ± 0.13	0.33 ± 0.03	81.89 ± 2.76	93.07 ± 0.37
HI cells-A55	12.76 ± 0.59	11.82 ± 0.06	0.34 ± 0.02	75.29 ± 2.15	95.97 ± 0.35
HI spores-A55	12.83 ± 0.25	10.56 ± 0.09	0.33 ± 0.05	80.19 ± 0.78	92.29 ± 1.64

Values are means ± standard deviation (SD) of triplicate assays. No statistically significant difference was found for each quality parameter. CK_1_* apple juice without PAT and *Alicyclobacillus* strains; CK_2_* (Control) PAT contaminated apple juice without *Alicyclobacillus* strains.

**Table 2:** Kinetic parameters for adsorption of PAT by the heat-inactivated (HI) cells and spores of *Alicyclobacillus acidocaldarius* DSM 451 (A51) at different temperatures and initial PAT concentrations.

Adsorbent	-	Pseudo-First Order Kinetic Model				Pseudo-Second Order Kinetic Model		
	Parameters	$q_{e}(\mu g/g)$	$k_{f}(h-1)$	$q_{e,cal(\mu g/g)}$	$R_{1}^{2}$	$k_{s}(g/\mu g\;h)$	$q_{e,cal(\mu g/g)}$	$R_{2}^{2}$
**HI cells-A51**	Temperature (°C)							
	20	11.426	0.052	13.144	0.950	0.0029	23.951	0.753
	30	12.621	0.069	16.995	0.999	0.0048	24.541	0.892
	40	12.203	0.062	16.014	0.982	0.0037	24.236	0.930
	Initial PAT concn. (μg/L)							
	50	4.211	0.098	6.423	0.918	0.019	5.681	0.901
	100	7.512	0.075	9.510	0.980	0.0073	11.415	0.830
	150	9.309	0.089	11.142	0.943	0.0063	12.725	0.911
	200	12.621	0.069	16.995	0.999	0.0048	24.541	0.892
	250	12.403	0.060	14.817	0.962	0.0025	21.426	0.745
**HI spores-A51**	Temperature (°C)							
	20	10.652	0.051	13.144	0.924	0.0022	16.304	0.628
	30	11.751	0.063	14.971	0.998	0.0038	23.985	0.873
	40	11.402	0.060	14.325	0.959	0.0025	23.457	0.901
	Initial PAT concn. (μg/L)							
	50	2.753	0.099	5.315	0.901	0.0140	5.025	0.814
	100	5.378	0.061	7.361	0.968	0.0049	10.950	0.733
	150	8.140	0.074	10.78	0.953	0.0051	11.862	0.913
	200	11.751	0.057	14.971	0.998	0.0038	23.985	0.873
	250	11.541	0.051	13.781	0.925	0.0022	21.056	0.719

**Table 3:** Adsorption isotherm parameters for patulin (PAT) on the heat-inactivated (HI) cells and spores of *Alicyclobacillus acidocaldarius* DSM 451 (A51).

Adsorbents	Langmuir Isotherm Model				Freundlich Isotherm Model		
	Temperature (°C)	$q_{m}(\mu g/g)$	$K_{L}(1/\mu g)$	$R^{2}$	$K_{F}(g/\mu g)({L/\mu g)}^{1/n})$	1/*n*	$R^{2}$
	20	11.759	1.214	0.9035	0.147	0.254	0.9991
**HI cells-A51**	30	12.755	1.356	0.9462	0.259	0.398	0.9998
	40	12.207	1.213	0.9119	0.201	0.303	0.9992
	20	10.956	1.031	0.8901	0.110	0.241	0.9990
**HI spores-A51**	30	11.823	1.244	0.9052	0.137	0.364	0.9996
	40	11.710	1.185	0.8642	0.131	0.301	0.9991

## Supplementary Materials

The following supplementary figures and tables are available online at http://www.mdpi.com/2072-6651/10/9/344/s1, Figure S1: Biomass of seven *Alicyclobacillus* strains. Each bar represents mean value of triplicate assays, error bars represent standard deviation and are significantly different based on one-way ANOVA with *P* < 0.05, Figure S2: A calibration standard curve of PAT, Figure S3. A representative standard chromatogram of patulin (PAT) (200 μg/L), Table S1: Relative cell viability and cytotoxicity rating scale, Table S2: Comparison of patulin (PAT) absorption by different adsorbents, Table S3: Standard *Alicyclobacillus* strains and their cultivation conditions, Table S4: Conditions used for adsorption of patulin (PAT) by HI cells and spores of *Alicyclobacillus* strains.
