# Supplementary material for: Effective Adsorption of Patulin from Apple Juice by Using Non-Cytotoxic Heat-Inactivated Cells and Spores of Alicyclobacillus Strains
Source: Toxins (Basel). 2018 Aug 25;10(9):344. doi: 10.3390/toxins10090344 (PMC6162514; doi:10.3390/toxins10090344)
Supplement: Supplementary file 1 [file toxins-10-00344-s001.pdf]

Supplementary Materials: Effective Adsorption of Patulin from Apple Juice by Using Non-cytotoxic Heat-inactivated Cells and Spores of *Alicyclobacillus* Strains

Marina Sajid, Sajid Mehmood, Chen Niu, Yahong Yuan and Tianli Yue

1. Results

Biomass of Seven Alicyclobacillus Strains

The biomass of the each *Alicyclobacillus* strain was compared as shown in Figure S1. The significant differences in biomass ($P < 0.05$) were found among these strains. *Alicyclobacillus pomorum* DSM 14955 (A55) strain produced the highest biomass (0.37 g/L), which was 12 times more than that of the lowest producer *Alicyclobacillus acidoterrestris* DSM 3923 (A23) with 0.03 g/L.

2. Materials and Methods

2.1. Determination of Bacteria Biomass

Alicyclobacillus strains were cultured independently into 150 mL *Alicyclobacillus acidocaldarius* medium (AAM) broth and incubated at 45°C for 24 h (150 rpm). After the first incubation, 3 mL of each activated *Alicyclobacillus* culture was transferred into a new AAM broth (150 mL) for enrichment incubation under the same conditions. Subsequently, the second culture liquid was centrifuged at 3600 *g* for 10 min and each bacterial paste was washed five times with physiological saline (0.85%, *w/v*) and then air-dried to constant weight at 60°C. The process was repeated three times. The biomass of each bacterial strain was calculated with the following equation: Biomass (g/L) = bacterial dry weight/culture volume.

3. Figures and Table Captions

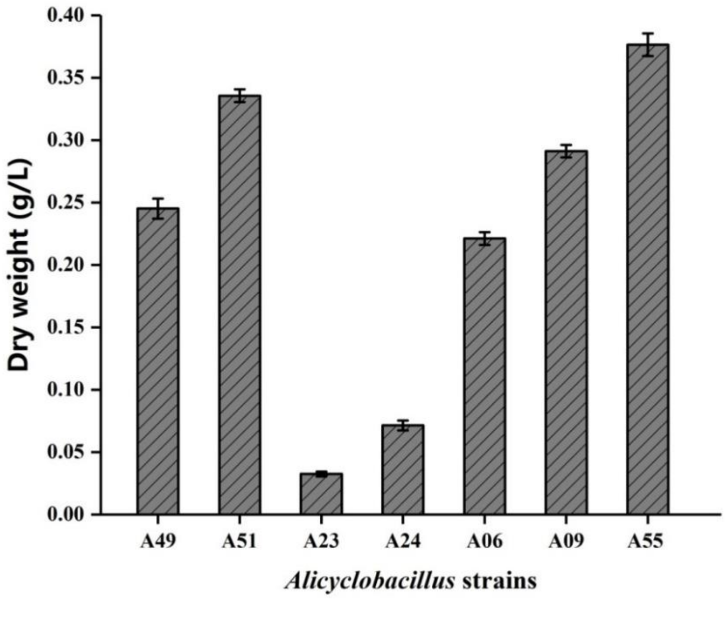

Figure S1. Biomass of seven *Alicyclobacillus* strains. Each bar represents mean value of triplicate assays, error bars represent standard deviation and are significantly different based on one-way ANOVA ($P < 0.05$).

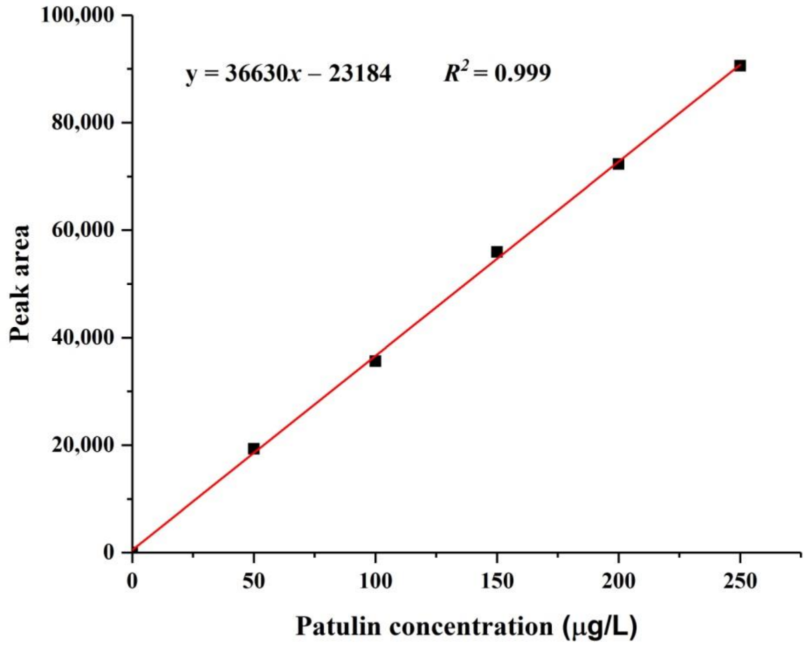

Figure S2. A calibration standard curve of patulin (PAT).

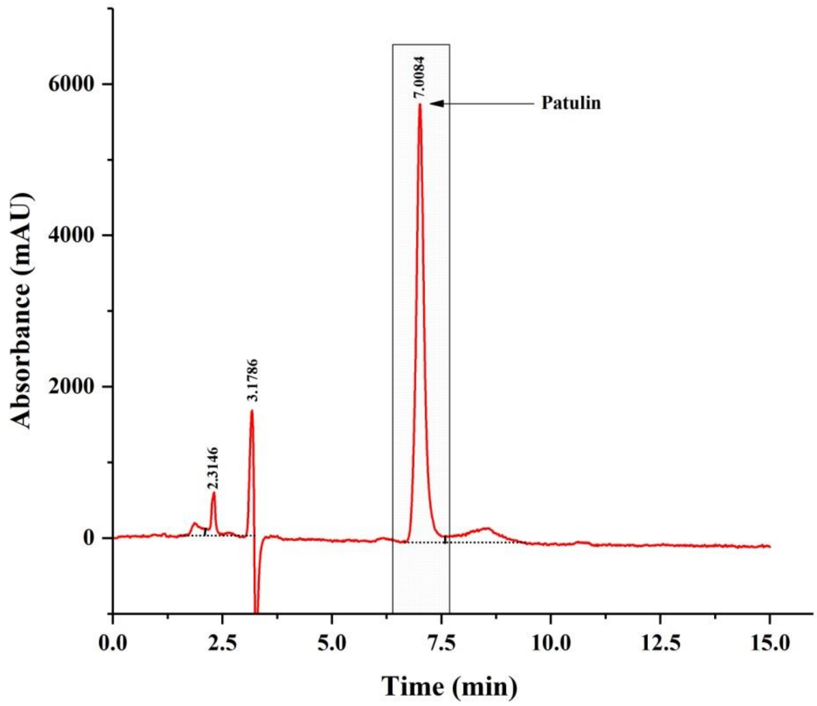

Figure S3. A representative standard chromatogram of patulin (PAT) (200 μg/L).

Table S1. Relative cell viability and cytotoxicity rating scale (0–5) [1,2].

Cell Viability (%)	Toxicity Rating Scale
≥ 100	0
75–99	1
50–74	2
25–49	3
1–24	4
0	5

Table S2. Comparison of patulin (PAT) absorption by different adsorbents.

Adsorbents	Absorbability	Adsorption time	Solvents	Reference
Inactive yeast YS3	1.36 μg/g	24 h	Apple juice	[3]
Propylthiol functionalized SBA-15	4 mg/g	24 h	Aqueous solution	[3]
Ca-alginate-AC beads	85 μg/g	5 h	Apple juice	[5]
Cross-link chitosan beads	626.4 μg/g	24 h	Aqueous solution	[6]
Thiourea modified chitosan resin	1.0 mg/g	24 h	Aqueous solution	[7]
Cross-linked xanthated chitosan	23.8 mg/g	14 h	Apple juice	[8]
Inactive yeast	11.55 μg/g	24 h	Kiwi fruit juice	[9]
Chitosan/Fe_3O_4 particles	19.4 μg/g	9 h	Kiwi fruit juice	[10]
Alicyclobacillus HI-cells A51	12.6 μg/g	24 h	Apple juice	This study
Alicyclobacillus HI-spores A51	11.8 μg/g	24 h	Apple juice	This study

Table S3. Standard *Alicyclobacillus* strains and their cultivation conditions.

		Cultivation conditions			
Alicyclobacillus **Species**	**Strains**	**Code**	**Medium**	**Temperature**	**pH**
Alicyclobacillus acidocaldarius	DSM 449	A49	AAM[A]	60 °C	3–4
Alicyclobacillus acidocaldarius	DSM 451	A51	AAM	60 °C	3–4
Alicyclobacillus acidoterrestris	DSM 3923	A23	AAM	60 °C	3–4
Alicyclobacillus acidoterrestris	DSM 3924	A24	AAM	60 °C	3–4
Alicyclobacillus cycloheptanicus	DSM 4006	A06	AAM	45 °C	4.5
Alicyclobacillus herbarius	DSM 13609	A09	AAM[B]	55 °C	4.2
Alicyclobacillus pomorum	DSM 14955	A55	AAM	45 °C	4.5

AAM (*Alicyclobacillus acidocaldarius* medium); Superscripts indicate the composition of AAM medium used for cultivation of *Alicyclobacillus* strains. AAM[A]: 2.0 g yeast extract powder, 5.0 g D-glucose, 0.2 g $(NH_4)_2SO_4$, 3.0 g KH_2PO_4, 0.5 g $MgSO_4.7H_2O$ and 0.25 g $CaCl_2$/L of deionizedwater (DI water: pH 4.0). AAM[B]: 0.2 g $(NH_4)_2SO_4$, 0.25 g $CaCl_2$, 0.6 g KH_2PO_4, 0.5 g $MgSO_4.7H_2O$, 0.01 g $MnSO_4$, 1.0 D-glucose and 2.0 g yeast extract powder per liter of deionized water (DI water: pH 3.0–4.0). AAM agar was prepared by adding 15–20 g agar per liter of AAM broth.

Table S4. Conditions used for adsorption of patulin (PAT) by HI cells and spores of *Alicyclobacillus* strains.

Parameters	Variables	Other Process Conditions
Incubation time (h)	0, 3, 6, 12, 18, 24, 36, 48	Temp. 30°C, agitation 120 rpm, pH 4.0, PAT concn. 200 μg/L
Temperature (°C)	20, 30, 40	Incubation time 24 h, agitation 120 rpm, pH 4.0, PAT concn. 200 μg/L
Initial Ph	2.0, 3.0, 4.0, 5.0, 6.0	Temp. 30°C, Incubation time 24 h, agitation 120 rpm, PAT concn. 200 μg/L
Initial PAT concn. (μg/L)	50, 100, 150, 200, 250	Temp. 30°C, Incubation time 24 h, agitation 120 rpm, pH 4.0, PAT concn. 200 μg/L

References

1. Zhang, D.; Du, Y. The biocompatibility study of Fe_3O_4 magnetic nanoparticles used in tumor hyperthermia. In Proceedings of 1st IEEE International Conference on Nano/Micro Engineered and Molecular Systems, Zhuhai, China, 18–21 January 2006; pp. 339–342.
2. Cheng, J.; Wu, W.W.; Chen, B.A.; Gao, F.; Xu, W.L.; Gao, C.; Ding, J.H.; Sun, Y.Y.; Song, H.H.; Bao, W.; et al. Effect of magnetic nanoparticles of Fe_3O_4 and 5-bromotetrandrine on reversal of multidrug resistance in K562/A02 leukemic cells. *Int. J. Nanomed.* **2009**, *4*, 209–216.
3. Yue, T.; Dong, Q.; Guo, C.; Worobo, R.W. Reducing patulin contamination in apple juice by using inactive yeast. *J. Food Prot.* **2011**, *74*, 149–153.
4. Appell, M.; Jackson, M.A., Dombrink-Kurtzman, M.A. Removal of patulin from aqueous solutions by propylthiol functionalized SBA-15. *J. Hazard. Mater.* **2011**, *187*, 150–156.
5. Yue, T.; Guo, C.; Yuan, Y.; Wang, Z.; Luo, Y.; Wang, L. Adsorptive removal of patulin from apple juice using Ca-alginate-activated carbon beads. *J. Food Sci.* **2013**, *78*, 1629- 1635.
6. Li, Y.; Wang, J.; Meng, X.; Liu, B. Removal of patulin from aqueous solution using cross-linked chitosan beads. *J. Food Saf.* **2015**, *35*, 248–256.
7. Liu, B.; Peng, X.; Chen, W.; Li, Y.; Meng, X.; Wang, D.; Yu, G. Adsorptive removal of patulin from aqueous solution using thiourea modified chitosan resin**.** *Int. J. Biol. Macromol.* **2015**, *80*, 520–528.
8. Peng, X.; Liu, B.; Chen, W.; Li, X.; Wang, Q.; Meng, X.; Wang, D. Effective biosorption of patulin from apple juice by cross-linked xanthated chitosan resin. *Food Control* **2016**, *63*, 140–146.
9. Luo, Y.; Wang, Z.L.; Yuan, Y.H.; Zhou Z.K.; Yue, T.L. Patulin adsorption of a superior microorganism strain with low flavour-affection of kiwi fruit juice. *World Mycotoxin J.* **2016**, *9*, 195–203.
10. Luo, Y.; Li, Z.; Yuan, Y.; Yue, T. Bioadsorption of patulin from kiwi fruit juice onto a superior magnetic chitosan. *J. Alloys Compd.* **2016**, *667*, 101–108.

© 2018 by the authors. Submitted for possible open access publication under the terms and conditions of the Creative Commons Attribution (CC BY) license (http://creativecommons.org/licenses/by/4.0/).
